# Molecular Detection of Metallo-β-Lactamase-Encoding Genes in the Clinical Isolates of Acinetobacter baumannii: A Prospective Cross-Sectional Study at a Tertiary Care Center

**DOI:** 10.7759/cureus.114100

**Published:** 2026-08-07

**Authors:** Anukriti Gupta, Navaneetha Chintala, Jyothi Lakshmi

**Affiliations:** 1 Microbiology, Gandhi Medical College, Secunderabad, IND

**Keywords:** acinetobacter baumannii, blandm, carbapenem-resistant acinetobacter baumannii (crab), metallo-β-lactamase (mbl), multidrug resistance (mdr)

## Abstract

Introduction: The rise of multidrug-resistant *Acinetobacter baumannii*, particularly in healthcare settings, poses significant treatment challenges. Detecting metallo-β-lactamase (MBL)-encoding genes in these isolates is crucial for understanding resistance mechanisms and guiding effective infection control strategies.

Objectives: The study aims (i) to evaluate the antibiotic susceptibility and resistance profiles of *Acinetobacter baumannii *and (ii) to detect MBL-encoding genes in multidrug-resistant isolates using multiplex polymerase chain reaction (PCR).

Materials and methods: A prospective cross-sectional study was conducted over 18 months (January 2023 to June 2024) at a tertiary care center. Ninety-four nonduplicate* Acinetobacter baumannii* isolates from various clinical samples collected from wards and ICUs were identified and tested for antimicrobial susceptibility using the VITEK 2 system (bioMérieux, Marcy-l'Étoile, France). PCR was used to identify the blaOXA-51-like gene, and all 94 nonduplicate isolates were screened for MBL genes (blaVIM, blaIMP, blaNDM, and blaGIM) using multiplex PCR.

Results: Of the 94 clinical *Acinetobacter baumannii *isolates, 41.48% were from ICU and emergency units. High resistance rates were observed for cephalosporins (92.56%) and carbapenems (imipenem 78.72%; meropenem 84.04%). A total of 79 (84.04%) isolates were identified phenotypically as carbapenem-resistant *Acinetobacter baumannii* (CRAB), all of which were positive for the blaOXA-51 gene. PCR screening of all 94 isolates revealed the presence of MBL-encoding genes in 68 isolates (72.34%), with blaNDM detected in 61 (64.89%), blaIMP in 11 (11.70%), blaVIM in six (6.38%), and blaGIM in four (4.25%) isolates. Coexistence of multiple MBL genes was observed in 11 (11.70%) isolates, predominantly the blaNDM + blaIMP combination in four (4.25%) isolates. A rare combination of blaNDM + blaVIM + blaIMP was identified in three isolates.

Conclusion: 72.34% of the total 94 *Acinetobacter baumannii* isolates harbored MBL genes, primarily blaNDM (61/94, 64.89%). The coexistence of multiple MBL genes in device-associated infections underscores the need for early detection to prevent transmission and reduce mortality. Prudent antibiotic use, continuous surveillance, and robust stewardship are essential for controlling MBL resistance. Given that this study was conducted at a single tertiary care facility, further research is needed to validate these findings in diverse healthcare settings.

## Introduction

*Acinetobacter baumannii*, an opportunistic Gram-negative bacterium, has become a predominant cause of healthcare-associated infections worldwide [1]. The World Health Organization (WHO) in 2017 listed *Acinetobacter baumannii *among critical antibiotic-resistant "priority pathogens," highlighting its serious threats to public health [2]. Plasmid-mediated metallo-β-lactamases (MBLs) like IMP, VIM, and NDM-1 further contribute to resistance and are critical due to their dissemination through integron-borne mobile gene cassettes [3-5]. The present study was undertaken to determine the prevalence of MBL-encoding genes (blaNDM, blaVIM, blaGIM, blaIMP) in the isolates of *Acinetobacter baumannii *in our hospital and their antibiotic sensitivity pattern so that appropriate infection control strategy and antibiotic policy can be formulated to prevent their spread.

## Materials and methods

This study was conducted at the Microbiology Department of Gandhi Medical College, a tertiary care teaching hospital in Secunderabad, India, from January 2023 to June 2024. A total of 94 consecutive nonduplicate isolates (first isolate from each patient during the study period) of *Acinetobacter baumannii *from different clinical samples (blood, respiratory sample, pus, urine, sterile body fluids, and other samples) were included in the study. Clearance was obtained from the Institutional Ethics Committee of Gandhi Medical College (approval number: IEC/GMC/2022/11/13) before starting the study.

Isolation and identification of the organism

Identification of the organism was done by direct smear of the sample using Gram stain and conventional bacteriological culture methods. Resistance to carbapenems was assessed using imipenem and meropenem discs on Mueller-Hinton agar. Isolates showing resistance to either carbapenem were categorized as carbapenem-resistant *Acinetobacter baumannii *(CRAB).

Further species-level identification and antibiotic susceptibility testing of the clinical isolates of* Acinetobacter baumannii *complex were performed using the VITEK 2 Compact system (bioMérieux, Marcy-l'Étoile, France) with VITEK GN identification cards and GN AST 406 cards. Colistin susceptibility was determined using the VITEK 2 Compact system. Confirmatory broth microdilution was not performed. The blaOXA-51-like gene, which is intrinsic to *Acinetobacter baumannii*, was detected using polymerase chain reaction (PCR). Susceptibility results were interpreted according to the Clinical and Laboratory Standards Institute (CLSI) M100 guidelines [6].

PCR amplification protocols

DNA extraction was done for all 94 isolates from overnight-grown colonies on nutrient agar using the HiPurA® bacterial genomic DNA purification kit (HiMedia Laboratories, Thane, India). Using the Bio-Rad T100 thermal cycler (Hercules, California, United States) and SimpliAmp thermal cycler (Applied Biosystems, Waltham, Massachusetts, United States), the primer sequences and cycling conditions are listed in Tables 1-3 [7,8]. All primers are procured from Bioserve Biotechnologies (India) Private Ltd. (Hyderabad, India)*. Acinetobacter baumannii *ATCC 19606 was used as a positive control for blaOXA-51, *Klebsiella pneumoniae* BAA 2146 was used as a positive control for blaNDM, and *Escherichia coli* NCTC 13476 was used as a positive control for blaIMP, respectively. Sterile nuclease-free water was used as the negative control.

**Table 1:** Uniplex polymerase chain reaction for the detection of the blaOXA-51-like gene

Gene	Primer sequence (5′-3′)	Amplicon size
blaOXA-51	F: TAATGCTTTGATCGGCCTTG	353 bp
	R: TGGATTGCACTTCATCTTGG	

**Table 2:** Thermocycler conditions for uniplex polymerase chain reaction

Procedure	Time
Initial denaturation	95°C for 15 minutes
DNA denaturation	95°C for 45 seconds
Annealing	57°C for 45 seconds
Primer extension	72°C for 1 minute
Final extension	72°C for 10 minutes

**Table 3:** Multiplex polymerase chain reaction for the detection of metallo-β-lactamase-encoding genes

Gene	Forward primer (5′-3′)	Reverse primer (5′-3′)	Expected amplicon size (bp)
blaIMP	GGAATAGAGTGGCTTAAYTCTC	CCAAACYACTASGTTATCT	232
blaVIM	GATGGTGTTTGGTCGCATA	CGAATGCGCAGCACCAG	390
blaGIM	TCGACACACCTTGGTCTGAA	AACTTCCAACTTTGCCATGC	477
blaNDM	GGTTTGGCGATCTGGTTTTC	CGGAATGGCTCATCACGATC	621

The thermal cycling conditions for multiplex PCR included an initial denaturation at 94°C for five minutes, followed by 30 cycles of DNA denaturation at 94°C for 30 seconds, annealing at 52°C for 40 seconds, and primer extension at 72°C for 50 seconds with a final extension step of 72°C for five minutes.

PCR products were examined through gel electrophoresis on a 2% agarose gel and visualized using the Vilber gel documentation system (Paris, France). Data were analyzed through a statistical analysis performed using Microsoft Excel (Microsoft Corporation, Redmond, Washington, United States). 

## Results

The demographic distribution showed a predominance of patients aged 41-60 years (31.91%), followed by those aged 21-40 years (29.79%) and under 20 years (20.21%). Gender distribution indicated a higher proportion among males (60.63%) compared to females (39.36%).

A total of 94 clinical isolates were obtained from various samples including blood, urine, sputum, and wound swabs. The highest proportion was found in 53 pus samples (56.38%), followed by 19 sputum samples (20.21%). ICU and emergency units reported the highest incidence (41.48%), with the acute medical care unit (AMC) having the maximum load among ICUs, followed by surgical (40.42%) and medical units (18.08%).

**Figure 1:**
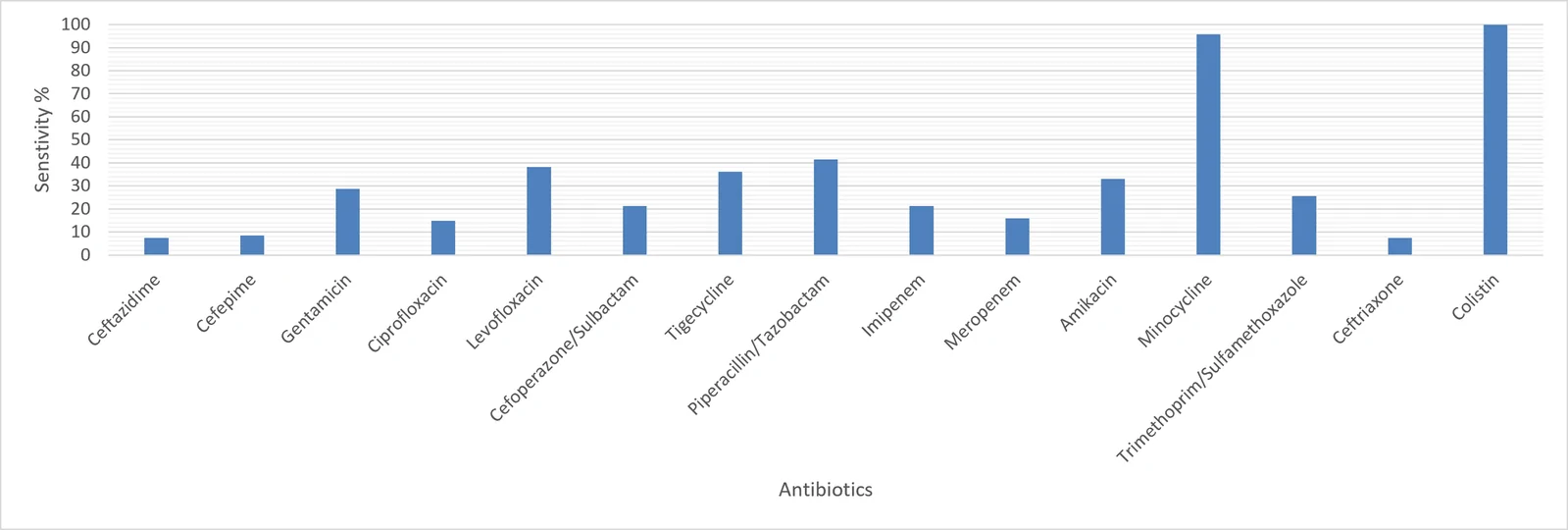
Antibiotic susceptibility pattern in the clinical isolates of Acinetobacter baumannii Antibiotic sensitivity profile of clinical *Acinetobacter baumannii *infection isolates (n=94) against commonly used antimicrobial agents. Highest sensitivity was observed for colistin (100%) and minocycline (95.74%), followed by piperacillin/tazobactam (41.48%) and tigecycline (36.17%). Values above the bars represent percentage sensitivity, while values within the bars indicate the number of sensitive isolates.

Antibiotic susceptibility testing revealed high resistance rates against ceftazidime (92.56%), cefepime (90.42%), and ciprofloxacin (82.98%), while colistin demonstrated 100% sensitivity and minocycline showed high efficacy (95.74%) (Figure 1).

Of the total isolates, 79 were identified as CRAB, all of which tested positive for the blaOXA-51 gene. Genotypic detection of MBLs revealed that 72.34% of all 94 isolates harbored at least one MBL gene. Genes were detected in a significant proportion of isolates: New Delhi MBL (NDM) in 61 (64.89%), imipenemase (IMP) in 11 (11.70%), Verona Integron-encoded MBL (VIM) in six (6.38%), and German imipenemase (GIM) in four (4.25%) samples (Table 4).

**Table 4:** Distribution of intrinsic blaOXA-51 and MBL-encoding genes detected by polymerase chain reaction among clinical Acinetobacter baumannii infection isolates (n=94) All isolates were positive for the intrinsic blaOXA-51 gene, confirming species identification as *Acinetobacter baumannii*.

Gene	blaOXA-51	blaNDM	blaVIM	blaGIM	blaIMP
Present	94 (100%)	61 (64.89%)	6 (6.38%)	4 (4.25%)	11 (11.70%)
Absent	0 (0%)	33 (35.11%)	88 (93.62%)	90 (95.75%)	83 (88.30%)

Among the clinical specimen types, pus-derived isolates accounted for the largest proportion of MBL-positive isolates, with blaNDM detected in 38/94 (40.43%), followed by blaIMP in 5/94 (5.32%), blaVIM in 4/94 (4.26%), and blaGIM in 1/94 (1.06%). Among sputum-derived isolates, blaNDM was detected in 9/94 (9.57%), followed by blaIMP in 2/94 (2.13%).

Further analysis of MBL genes revealed 11.70% of isolates showing coexistence of multiple MBL genes within individual samples, with NDM + IMP being the most prevalent (4.25%) as shown in Table 5.

**Table 5:** Coexistence of metallo-β-lactamase-encoding genes in various clinical samples (n=94)

Genes detected	No. of samples	%
blaNDM + blaIMP	4	4.25%
blaNDM + blaVIM	2	2.12%
blaNDM + blaGIM	2	2.12%
blaNDM + blaVIM + blaIMP	3	3.19%

**Figure 2:**
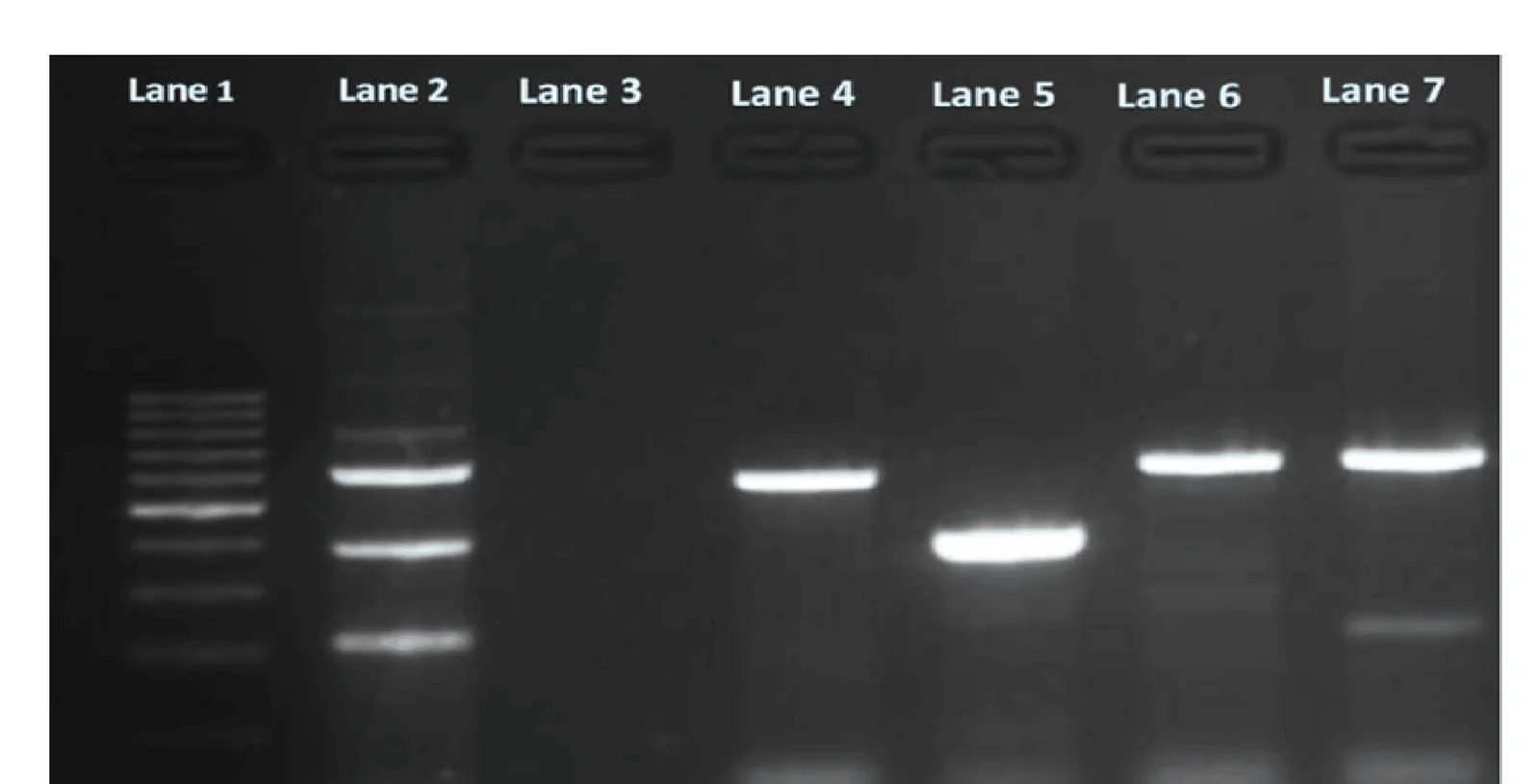
Gel documentation image of multiplex polymerase chain reaction Lane 1: 100 bp ladder. Lane 2: positive control (NDM: 621 bp; VIM: 390 bp; IMP: 232 bp). Lane 3: negative control. Lane 4: NDM (621 bp) positive clinical sample. Lane 5: VIM (390bp) positive clinical sample. Lane 6: NDM (621 bp) positive clinical sample. Lane 7: NDM (621bp) and IMP (232 bp) positive clinical sample. bp: base pairs; IMP: imipenemase; VIM: Verona Integron-encoded metallo-β-lactamase; GIM: German imipenemase; NDM: New Delhi metallo-β-lactamase

Three MBLs (blaIMP, blaVIM, and blaNDM) were found to coexist in three isolates (Figure 2).

## Discussion

The study highlighted that patients aged over 40 years (31.91%) and predominantly male (60.63%) showed a greater proportion of* Acinetobacter baumannii* infections. Similar findings were reported by Wajid et al. [9] and other studies showing male predominance up to 64.4%. Risk factors identified included previous hospitalization (17.02%), surgery (13.82%), and pulmonary disease (13%), consistent with previous research [10-12]. Infection incidence was highest in ICU and emergency units (41.48%), followed by surgical units (40.42%) and medical units (18.08%), consistent with previous observations [13]. Pus samples (56.38%) were the most common source, aligning with a study done by Madhavi et al. [14].

The study also reported high resistance rates against third-generation cephalosporins (up to 92.56%) and moderate resistance to antibiotics like gentamicin, ciprofloxacin, and cefoperazone/sulbactam (60-85%). Minocycline and piperacillin-tazobactam showed lower resistance and demonstrated favorable in vitro activity. Colistin demonstrated universal sensitivity. Standard treatment options for CRAB are limited. Combination therapy is given preference over monotherapy. Minocycline, tigecycline, and colistin are recommended due to lower minimum inhibitory concentration (MIC) and established efficacy [15,16]. These findings emphasize the urgent need for alternative treatment strategies amidst rising multidrug-resistant infections.

A similar study conducted in South India reported a lower but significant rate of carbapenem resistance at 66% [17]. However, the distribution of multidrug-resistant *Acinetobacter baumannii* varies widely worldwide due to diverse environmental pressures, ranging from as low as 2.8% in Japan to as high as 88% in South Korea [16].

*Acinetobacter*-derived cephalosporinases and OXA-51-like enzymes are intrinsic β-lactamases encoded within the chromosomes of *Acinetobacter baumannii *[16]. In the present study, all 94 isolates tested positive for the OXA-51 gene, underscoring its ubiquitous presence among *Acinetobacter baumannii* strains. The prevalence of the MBL gene is similar to other studies [18-23] (Table 6). 

**Table 6:** Prevalence of MBL genes in Indian studies MBL: metallo-β-lactamase

S. no.	Study	Result
1	Sharma et al. [18]	MBL positivity ranges from 9.3% to 70.9% in India
2	Mahajan et al. [19]	66.7% MBL positivity, with blaVIM being the most common gene (49.3%), followed by blaNDM-1 (18.7%)
3	Vijayakumar et al. [20]	blaNDM is the predominant gene (21.28%)
4	Rynga et al. [21]	The most predominant gene is blaGIM (6%)
5	Santhiya et al. [22]	Presence of five different MBL genes in a single isolate
6	Girija et al. [23]	Co-occurrence of blaVIM and blaGIM
7	Present study	72.34% isolates positive for MBL genes. Predominant gene: blaNDM (64.48%). Coexistence: blaNDM with blaIMP, blaVIM, and blaGIM (11.70%). Coexistence in 11.70% (11/94) of isolates. Triple combination: blaNDM, blaVIM, and blaIMP found in three isolates

Limitations of the study

While this study offers valuable insights, it is essential to acknowledge several limitations. The research was conducted at a single tertiary care facility, which may restrict its generalizability to other healthcare settings. Whole-genome sequencing or allele-level confirmation (e.g., NDM-1 vs. NDM-5) could not be performed due to financial constraints, although gene-level specificity of primers was validated using reference strains and expected amplicon sizes. Colistin susceptibility was determined using the VITEK 2 Compact system and was not confirmed by the reference broth microdilution method. Future studies should seek to validate these findings across varied geographical locations and healthcare environments. Furthermore, longitudinal research is necessary to monitor resistance trends over time and evaluate the effectiveness of interventions aimed at managing *Acinetobacter baumannii* infections. Future research should explore additional mechanisms like efflux pumps to comprehensively understand carbapenem resistance in *Acinetobacter baumannii*.

## Conclusions

A concerning prevalence of 72.34% MBL-encoding genes was observed among the clinical isolates of *Acinetobacter baumannii*, with blaNDM being the predominant gene in 64.89% of isolates. The study incidentally identified the coexistence of multiple MBL genes in isolates from infections in respiratory and surgical units, highlighting the urgent need for early detection of MBL genes to lower mortality rates and prevent intra-hospital spread. Colistin and minocycline demonstrated favorable in vitro activity against the tested isolates. However, antimicrobial therapy should be guided by current clinical guidelines, antimicrobial susceptibility results, infection site, and patient-specific factors. Further multicenter studies incorporating comprehensive molecular characterization and clinical outcome data are warranted to validate these findings. Continuous surveillance of MBL prevalence and implementation of robust antibiotic stewardship programs are essential for the effective management and control of MBL resistance in healthcare settings.
